# *Taxus yunnanensis* genome offers insights into gymnosperm phylogeny and taxol production

**DOI:** 10.1038/s42003-021-02697-8

**Published:** 2021-10-20

**Authors:** Chi Song, Fangfang Fu, Lulu Yang, Yan Niu, Zhaoyang Tian, Xiangxiang He, Xiaoming Yang, Jie Chen, Wei Sun, Tao Wan, Han Zhang, Yicheng Yang, Tian Xiao, Komivi Dossa, Xiangxiao Meng, Fuliang Cao, Yves Van de Peer, Guibin Wang, Shilin Chen

**Affiliations:** 1 China Academy of Chinese Medical Sciences, Institute of Chinese Materia Medica, 100070 Beijing, China; 2 Co-Innovation Center for Sustainable Forestry in Southern China, College of Forestry, Nanjing Forestry University, 210037 Nanjing, Jiangsu China; 3 Department of Cell Biology and Genetics, Shenzhen University Health Sciences Center, 1066 Xueyuan Avenue, 518060 Shenzhen, Guangdong China; 4 Wuhan Benagen Tech Solutions Company Limited, 430070 Wuhan, Hubei China; 5 Sino-Africa Joint Research Center, Chinese Academy of Science, 430074 Wuhan, Hubei China; 6 Innovative Institute of Chinese Medicine and Pharmacy, Chengdu University of Traditional Chinese Medicine, 611137 Chengdu, Sichuan China; 7 CIRAD, UMR AGAP Institut, F-34398 Montpellier, France; 8 Department of Plant Biotechnology and Bioinformatics, Ghent University, Ghent, Belgium; 9 VIB Center for Plant Systems Biology, Ghent, Belgium; 10 Centre for Microbial Ecology and Genomics, Department of Biochemistry, Genetics and Microbiology Genetics, University of Pretoria, Private Bag X20, Pretoria, 0028 South Africa; 11 Academy for Advanced Interdisciplinary Studies and College of Horticulture, Nanjing Agricultural University, 210095 Nanjing, Jiangsu China; 12 Present Address: Innovative Institute of Chinese Medicine and Pharmacy, Chengdu University of Traditional Chinese Medicine, 611137 Chengdu, Sichuan China

**Keywords:** Plant evolution, Secondary metabolism

## Abstract

Taxol, a natural product derived from *Taxus*, is one of the most effective natural anticancer drugs and the biosynthetic pathway of Taxol is the basis of heterologous bio-production. Here, we report a high-quality genome assembly and annotation of *Taxus yunnanensis* based on 10.7 Gb sequences assembled into 12 chromosomes with contig N50 and scaffold N50 of 2.89 Mb and 966.80 Mb, respectively. Phylogenomic analyses show that *T. yunnanensis* is most closely related to *Sequoiadendron giganteum* among the sampled taxa, with an estimated divergence time of 133.4−213.0 MYA. As with most gymnosperms, and unlike most angiosperms, there is no evidence of a recent whole-genome duplication in *T. yunnanensis*. Repetitive sequences, especially long terminal repeat retrotransposons, are prevalent in the *T. yunnanensis* genome, contributing to its large genome size. We further integrated genomic and transcriptomic data to unveil clusters of genes involved in Taxol synthesis, located on the chromosome 12, while gene families encoding hydroxylase in the Taxol pathway exhibited significant expansion. Our study contributes to the further elucidation of gymnosperm relationships and the Taxol biosynthetic pathway.

## Abstract (web summary)

Song, Fu, Yang, Niu, Tian, et al. present a high-quality genome assembly and transcriptome data of Taxus yunnanensis, a plant belonging to the Taxus genus known for sources of traditional medicine and natural anticancer drugs. This study explores the Taxus evolutionary history and sheds light on the Taxol biosynthetic pathway.

## Introduction

*Taxus* species, belonging to the Taxaceae (yews, gymnosperms), are slow-growing, long-lived coniferous trees or shrubs, that have been regarded as endangered Tertiary relict species. *Taxus* are well-known for their cancer-inhibitory alkaloid paclitaxel (Taxol), which is a trace natural product^1^. Taxol is a polyoxygenated cyclic diterpenoid, mainly used to treat numerous cancers, including ovarian, breast, lung, cervical, and pancreatic cancer^2–5^. However, limited content (0.01–0.05 %) and localization of paclitaxel in specific organs (the bark of yew) renders production from natural sources low^3,6^. Taxol biosynthesis begins with the universal diterpenoid precursor geranylgeranyl diphosphate (GGPP), which is then decorated with a series of cytochrome-P450 hydroxylases (CYP450s), acyltransferases and other enzymes, leading to the end product paclitaxel^7,8^. The complexity of Taxol biosynthesis has greatly hindered the mass production of Taxol^3,9^.

To further elucidate the Taxol biosynthesis pathway, we here report a high-quality genome assembly of *T. yunnanensis*. In addition, the genome of *Taxus*, as a first representative of Taxaceae, might help in unraveling the phylogenetic relationships within gymnosperms. There is much controversy about the evolutionary relationship between different gymnosperms (such as Cycads, Ginkgo, Gnetophytes and Conifers)^10–12^. The 1 KP transcriptome dataset provides strong support for Cycads and Ginkgo sister to the rest of gymnosperms and Gnetophytes sister to, or within, the conifers^13–15^. Whole-genome sequences, such as the one of *Taxus* presented here, provide an additional dataset to shed light on the elusive evolutionary relationships within gymnosperms.

## Results and discussion

**Fig. 1:**
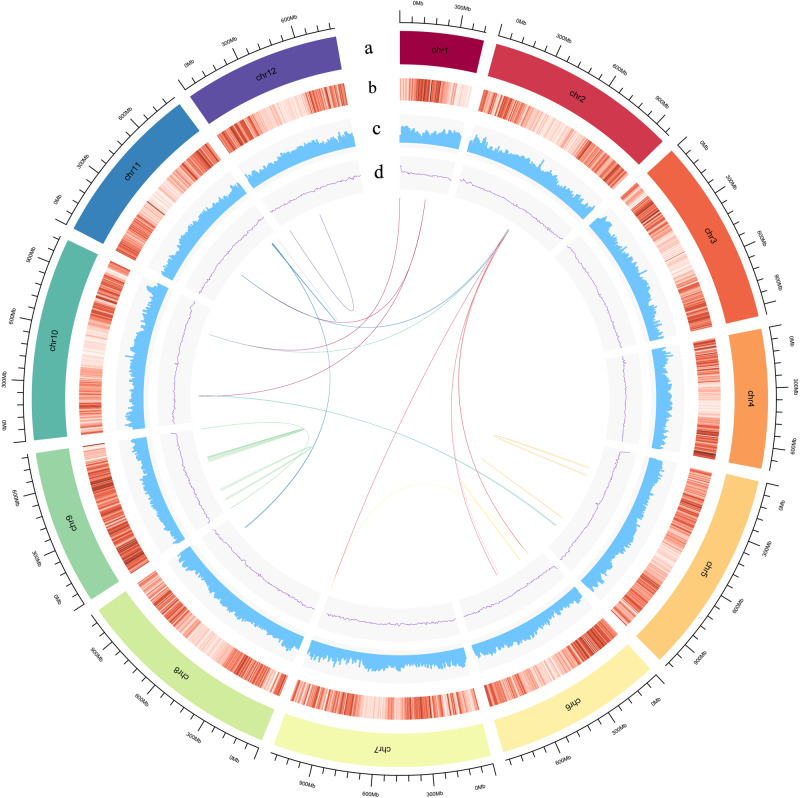
Distribution of *T. yunnanensis* genomic features. **a** Circular representation of the 12 pseudochromosomes, **b** gene density (5 Mb window), **c** percentage of repeats (5 Mb window), **d** GC content (5 Mb window), and intragenomic syntenic regions denoted by a single line represent a genomic syntenic region covering at least five paralogues.

Based on the k-mer distribution analysis, we estimated the genome size of *T. yunnanensis* to be 10.49 Gb, with a high level of repetition (77.74%) and heterozygosity (0.54%) (Supplementary Fig. 1 and Supplementary Table 1). The genome sequence of *T. yunnanensis* was obtained using Oxford Nanopore high-throughput sequencing systems (85×), Illumina (50×) and high-throughput chromosome conformation capture (Hi-C, 60×) (Supplementary Table 2). The total length of the final assembly was 10.73 Gb with a contig N50 of 2.89 Mb and a scaffold N50 of 966.80 Mb (Table 1 and Supplementary Table 3). A total of 10.63 Gb of the assembly and 98.95% of the genes were distributed across 12 chromosome-level pseudomolecules (Supplementary Table 3 and Supplementary Fig. 2). The completeness of the genome assembly and gene set of *T. yunnanensis* were estimated at 72.6% and 73.7% using BUSCO, which is similar to the available gymnosperm genomes (Supplementary Table 4)^16–18^. We annotated 34,931 high-quality protein-coding genes, which is slightly lower than for the *S. giganteum* genome (38,000)^17^ (Fig. 1 and Table 1). On average, the predicted gene sequence length was 21,831.74 bp, containing 4.58 exons with an average sequence length of 305.46 bp (Table 1). Numerous long introns are a notable characteristic of *T. yunnanensis* genome. The length distribution for the 10% longest introns in *T. yunnanensis* is from 14,790 bp to 462,177 bp, and average at 35,282 bp. A comparison of gene models for the 14 land plants revealed that the average length of the longest 10% of introns in most of the gymnosperms was longer than that in angiosperms (Supplementary Table 5 and Supplementary Fig. 3).

**Table 1:** Assembly and annotation statistics of the draft genome of *T. yunnanensis*.

Assembly features	
Total length of scaffolds (bp)	10,738,316,084
Longest scaffold (bp)	1,071,627,631
N50 of scaffold (bp)	966,801,426
Total length of contigs (bp)	10,737,203,084
Longest contig (bp)	22,834,067
N50 of contig (bp)	2,892,145
GC ratio (%)	36.91
Total number of contigs	11,280

Genome annotation	
Number of protein-coding genes	34,931
Average CDS length (bp)	910.80
Average exon/intron length (bp)	305.46/5702.27
Average exon per gene	4.58

A total of 7.96 Gb of repetitive elements occupying 74.11% of the *T. yunnanensis* genome were annotated (Supplementary Table 6). Repetitive sequences, especially the long terminal repeat retrotransposons (LTR-RTs), have been deemed to be the major component of all gymnosperm genomes and the main cause of gymnosperm genome expansion^12,16,17,19^. Consistent with other gymnosperm genomes, the majority of the repeats in the *T. yunnanensis* genome are LTR (40.95% of all assembled sequences), of which two super-families, 2,138,065 Ty3/Gypsy and 453,398 Ty1/Copia (the number of repeats sequences) were identified, accounting for 35.95% and 4.77% of all assembled sequences, respectively (Supplementary Table 6). Based on a mutation rate of 7.34573 × 10^−10^ substitutions per base per year, we found that the insertion for Gypsy and Copia occurred largely between 8−24 and 8−44 million years ago (MYA), respectively (Supplementary Fig. 4a). Since the Gypsy accounted for 87.78% of the total LTR sequences, the insertion of large amounts of Gypsy in 8−24 MYA resulted in genome expansion of *T. yunnanensis*. We identified and characterized full-length LTR in four gymnosperms and three angiosperms (*T. yunnanensis, Gnetum montanum*, *Ginkgo biloba*, *S. giganteum*, *Amborella trichopoda*, *Oryza sativa* and *Arabidopsis thaliana*), the number of LTRs contained in gymnosperms was higher than those in angiosperms (Supplementary Table 7). Phylogenetic reconstructions revealed that conifers displayed substantially higher diversity and abundance than *G. montanum* and *G. biloba*, possibly indicating gradual and/or rapid diversification in conifers (Supplementary Fig. 4b).

**Fig. 2:**
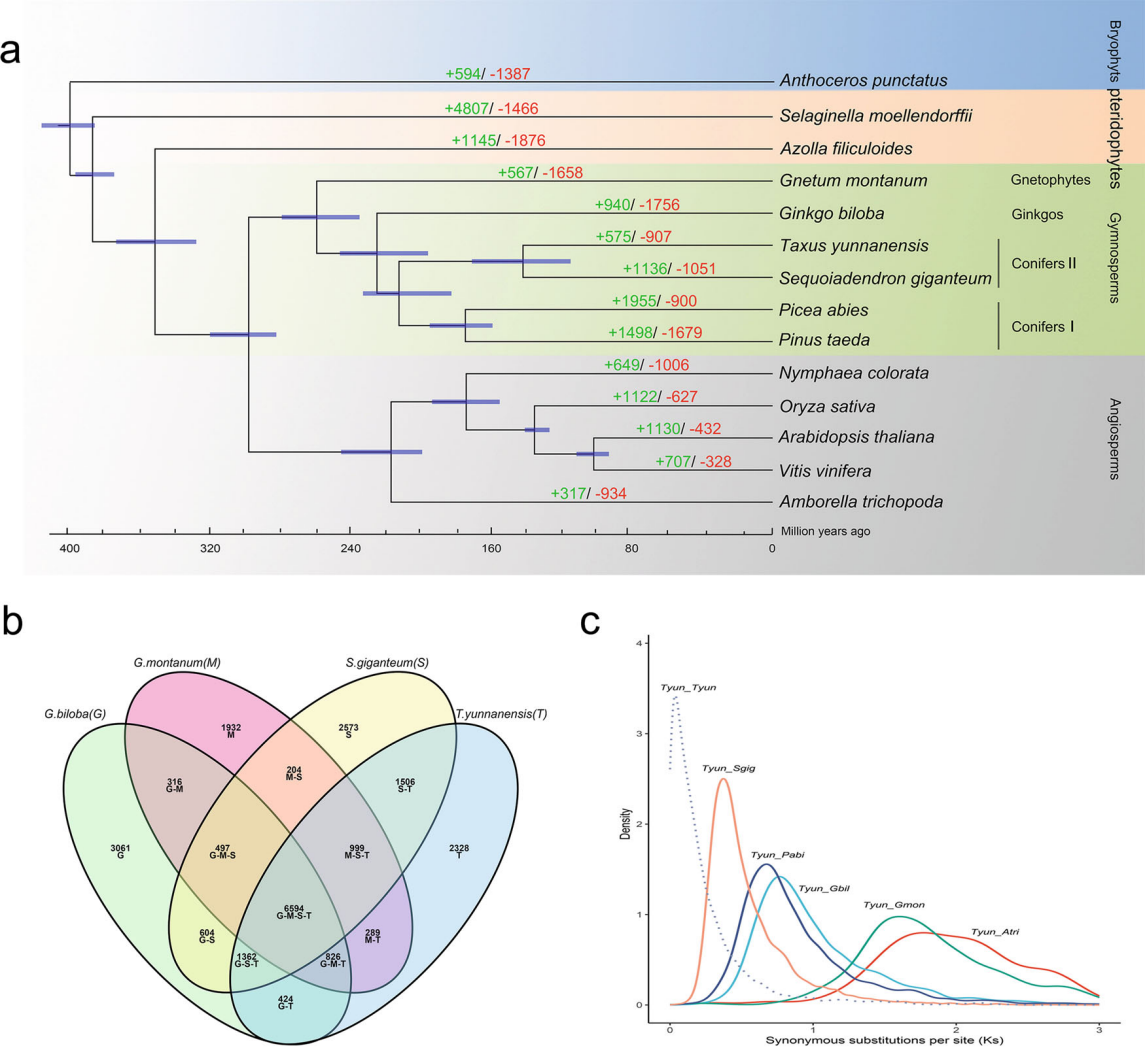
Genome evolution of *T. yunnanensis*. **a** Inferred phylogenetic tree with 588 single-copy gene families in 14 plant species. Gene family expansions are indicated in green, and gene family contractions are indicated in red. Blue bars at nodes represent divergence times estimated by Maximum Likelihood (PAML). **b** Shared and unique gene families in four species. **c** Synonymous substitutions per synonymous site (Ks) distributions of orthologous (and paralogous) genes between *T. yunnanensis* and *G. montanum*, *G. biloba*, *P. abies*, *S. giganteum* and *A. trichopoda*.

The *T. yunnanensis* genome, as a second member belonging to the so-called conifers II clade for which the genome sequence has been determined, provides an opportunity to revisit the relationships of gymnosperms. Using six gymnosperms, five angiosperms and two pteridophytes, and *Anthoceros punctatus* as an outgroup, we identified 588 single-copy gene families (5951 genes in all of the 14 species) to construct a phylogenetic tree, using ASTRAL and ‘supertree’ based on amino acid alignments, DNA alignments, codon alignments and codon alignments with third-positions removed (Fig. 2a, Supplementary Table 8 and Supplementary Data 1). All of the phylogenomic analyses showed that *T. yunnanensis* was most related to *S. giganteum*, with an estimated divergence time of 133.4−213.0 MYA, representing the conifers II clade. The split between conifers I and conifers II was estimated at 219.1−257.2 MYA. All but one of the ASTRAL analyses (DNA alignment) placed *G. montanum* as sister to all other extant gymnosperm lineages, further supporting the Gnetales-other gymnosperms hypothesis of gymnosperm phylogeny^10,20^ (Supplementary Fig. 5). However, this relationship is at odds with a general phylogeny proposed by the 1KP consortium^13^, which finds Cycad and Ginkgo as sisters to the rest of gymnosperms based on transcriptome data. This difference may require further study, such as the use of genome data for additional gymnosperms.

A total of 575 gene families were expanded, 55 of which exhibited significant expansion (*P* < 0.05), relative to the ancestor of *T. yunnanensis* and *S. giganteum* (Fig. 2a). Some of these genes were annotated as a cellular component, such as apoplast (GO:0048046) and nucleosome (GO:0000786); the biological process chromosome stability such as DNA integration (GO:0015074), telomere capping (GO:0016233) and mitotic cell cycle (GO:0000278); molecular functions related to the synthesis of the primary metabolite, such as aspartic-type endopeptidase activity (GO:0004190), cysteine-type peptidase activity (GO:0008234), polysaccharide binding (GO:0030247) and protein heterodimerization activity (GO:0046982) (Supplementary Fig. 6 and Supplementary Data 2). Seventy-two genes related to the apoplast, of which 57 genes were annotated as Dirigent protein in the UniProt database, which were discovered in coniferous trees, and participating in lignan biosynthesis for defense purposes (Supplementary Data 3). A total of 907 gene families, many involved in ATPase activity, coupled to transmembrane movement of substances (GO:0042626), ATPase activity (GO:0016887) and transmembrane transport (GO:0055085), showed contraction (Supplementary Fig. 7 and Supplementary Data 4).

Among *T. yunnanensis*, *S. giganteum*, *G. montanum*, and *G. biloba* gene families, a total of 2328 gene families appeared unique to *T. yunnanensis* (Fig. 2b and Supplementary Data 5), and were particularly enriched in isoquinoline alkaloid biosynthesis (ko00950), flavone and flavonol biosynthesis (ko00944), and ubiquinone and other terpenoid-quinone biosynthesis (ko00130) (Supplementary Fig. 8 and Supplementary Data 6).

Most angiosperms have undergone whole-genome duplication (WGD) somewhere during their evolutionary past. Although it has been reported that all seed plants shared an ancient WGD, WGDs in gymnosperms seem to be much rarer^21–23^. WGDs are usually identified from *Ks* (a measure of the number of substitutions per synonymous site) age distributions of paralogs, or from gene collinearity data. Since *Ks* age distributions showed no clear peaks and no widespread intragenomic colinear or syntenic segments could be detected, we assume no recent WGD event has occurred in the evolutionary past of *T. yunnanensis*, although older WGDs cannot be excluded (Fig. 2c, Supplementary Fig. 9 and Supplementary Fig. 10). Evidence for small-scale gene duplication events is more evident and general analysis of gene duplication in *T. yunnanensis* shows that dispersed duplicates (60.07%) from the dominant type compared to three other types: WGD/segmental duplication (0.75%), proximal (11.66%) and tandem (13.09%) (see ‘Methods’).

**Fig. 3:**
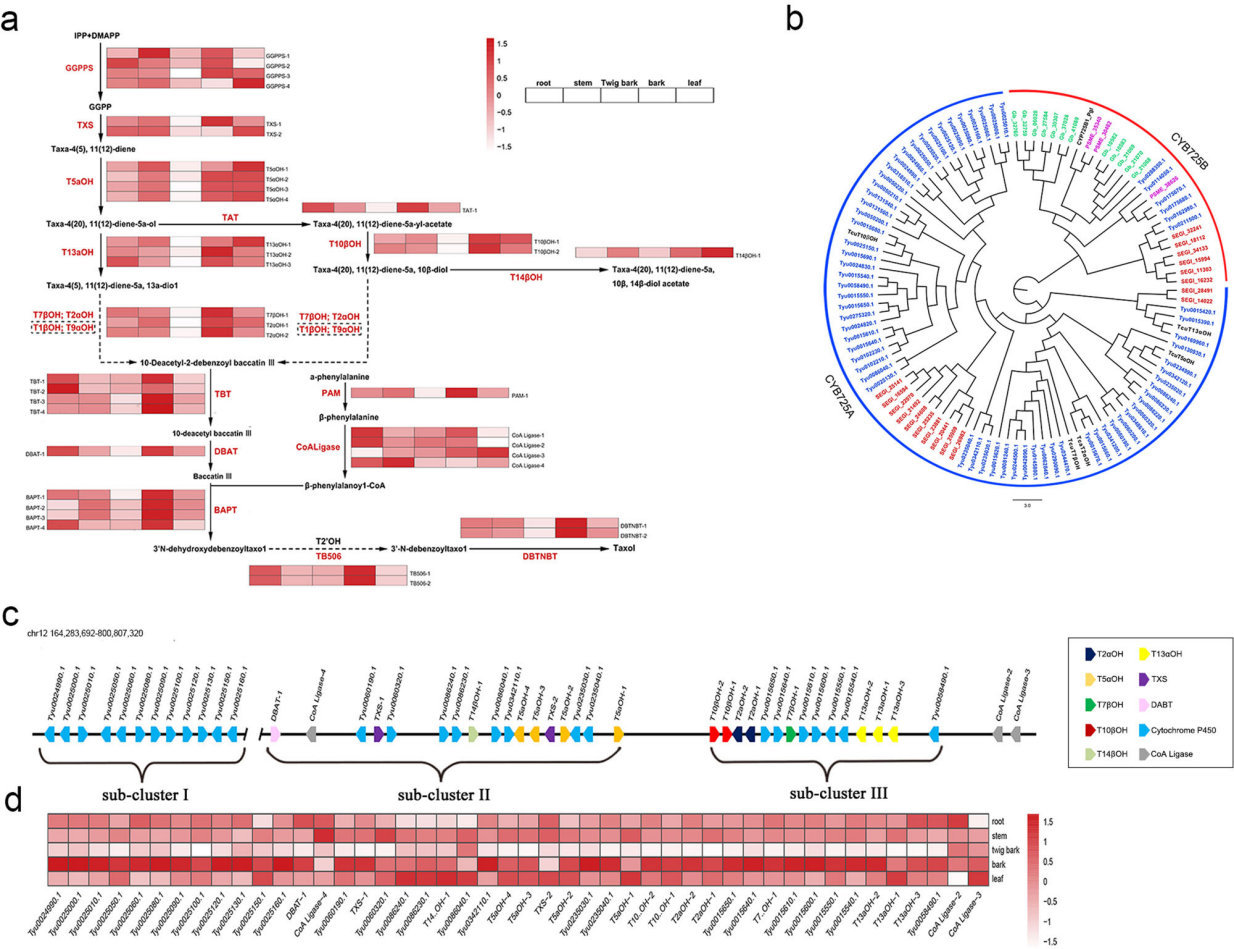
Genes involved in the Taxol biosynthetic pathway. **a** Transcriptomic analysis of genes involved in the Taxol biosynthetic pathway. The FPKM was calculated to evaluate the expression level of each gene. T1βOH and T9αOH represent the enzymes responsible for C-1 and C-9 oxidation that are currently unverified and which are presumed to belong to CYP450 gene family. **b** Phylogenetic tree of the CYP725A gene sub-family in *T. yunnanensis*, *S. giganteum*, *P. menziesii* and *G. biloba*. Genes from the four different plants are labeled in different colors, Blue, *T. yunnanensis*; Pink, *P. menziesii*; Red, *S. giganteum*; Green, *G. biloba*. **c** Arrangement and chromosomal positions of three Taxol gene clusters on chromosome 12 (chr12). **d** Heat maps of gene expression of CYP725A genes located on chromosome 12. The average expression profiles of three replicates of different tissues of *T. yunnanensis* were used to make the heat map. Color scale represents log2-transformed FPKM (expected number of fragments per kilobase of transcript sequence per millions base pairs sequenced) values. The gradual change of the color indicates the different expression levels of genes, white indicating low transcript abundance and red indicating high levels of transcript abundance.

All of the hydroxylases involved in Taxol biosynthesis belong to CYP450s^7^. The CYP450s responsible for hydroxylation at the C-2, C-5, C-7, C-10, C-13 and C-2ʹ positions have been characterized in *Taxus*^8,24–28^, while the enzymes responsible for C-1 and C-9 oxidation are currently unknown (Fig. 3a and Supplementary Data 7). Most of the enzymes, identified to be involved in Taxol biosynthesis, are encoded by multiple gene copies in *Taxus*, especially the CYP450s genes such as taxoid 10β–hydroxylase (T10βOH) and taxoid 5α–hydroxylase (T5αOH) (Fig. 3a and Supplementary Data 7).

So far, Taxol is found only in *Taxus* species, indicating that some of the CYPs involved in the biosynthesis of Taxol may be specific to *Taxus*. In order to identify such species-specific CYPs, we compared a total of 3368 CYP genes in *T. yunnanensis*, *S. giganteum*, *G. biloba*, *G. montanum*, *Picea glauca*, *Pseudotsuga menziesii* and *A. thaliana* (Supplementary Table 9). In total, 624 CYP450s genes were identified in *T. yunnanensis* genome and the number of CYP725 sub-family genes was substantially higher than that in other species (Supplementary Table 9). The CYP450s genes involved in the biosynthesis of Taxol belong to CYP725A sub-family^8^. We constructed a phylogenetic tree from CYP725 genes obtained from a genome sequence alignment of five species (*T. yunnanensis*, *S. giganteum*, *G. biloba*, *P. glauca* and *P. menziesii*) (Fig. 3b and Supplementary Fig. 11). Sixty-eight specific CYP725 genes were found in the *T. yunnanensis* genome, of which 62 genes belong to the CYP725A sub-family. Although few CYP725 genes have also been identified in the genomes of *S. giganteum* and *G. biloba*, only 12 CYP725 genes of *S. giganteum* belonged to two of the clades of the CYP725A sub-family, while all of 12 CYP725 genes of *G. biloba* belong to the CYP725B sub-family (Fig. 3b, Supplementary Fig. 10 and Supplementary Data 8).

The genome assembly allowed us to locate all of the functionally characterized genes of Taxol metabolism in *Taxus*, as well as their closely related homologs, on either the chromosome or the unplaced scaffold positions. Forty CYP725A genes were found distributed on chromosome 12, including hydroxylation-like genes responsible for C-2, C-5, C-7, C-10, C-13 and C-14 hydroxylation. Moreover, taxadiene synthase (TXS) and 10-deacetylbaccatin III-10-O-acetyltransferase (DBAT) like genes involved in the Taxol biosynthetic pathway were also located on chromosome 12 (Fig. 3c and Supplementary Data 9). These genes were grouped on a 76.2 Mb region to form a taxol synthesis gene cluster which was artificially divided into three sub-clusters (sub-cluster I, II, III) (Fig. 3c). We detected 12 functionally uncharacterized CYP725 genes in the sub-cluster I, which exhibited a similar expression pattern (Pearson correlation coefficient > 0.8, *P* < 0.05) with T5αOH, T10βOH, T2αOH, T7βOH and T13αOH, and were highly expressed in bark (Fig. 3c, d). We suspect that these genes may participate in the production of Taxol. TXS and T5αOH are encoded by co-localized gene copies in sub-cluster II; T10βOH, T2αOH, T7βOH and T13αOH are encoded by co-localized gene copies in sub-cluster III, which were all highly expressed in the bark of *T. yunnanensis* (Fig. 3c, d; Supplementary Data 9, 10). Moreover, 15 functionally uncharacterized CYP725 genes localized in sub-cluster II and III, which have low homology with known hydroxylation-like genes in Taxol pathway, while exhibiting high and similar expression patterns (Pearson correlation coefficient > 0.8, *P* < 0.05) as the known genes functioning in Taxol metabolism. These genes might be interesting as potential candidates genes in Taxol biosynthesis pathway (Supplementary Data 9, 10).

## Conclusions

This study reports a high-quality chromosome-level genome assembly for *T. yunnanensis*. This provides crucial information for the study of the evolution of gymnosperms. We estimated that there is no evidence of a recent WGD in *T. yunnanensis* and LTR expansion is the main cause of its large genome size. Interestingly, the CYP725A gene families, encoding hydroxylase involved in Taxol synthesis, exhibited significant expansion, and most of them clustered on chromosome 12 and exhibited co-expression, which contributes to the further elucidation of the Taxol biosynthetic pathway.

## Methods

### Plant materials, DNA library construction and sequencing

Fresh leaves were collected from *T. yunnanensis* in Yunnan province. High-quality genomic DNA was isolated from the fresh leaves using the CTAB method^29^, and the DNA quality and concentration were tested by 0.75% agarose gel electrophoresis, NanoDrop One spectrophotometer (Thermo Fisher Scientific) and Qubit 3.0 Fluorometer (Life Technologies, Carlsbad, CA, USA).

After the DNA quality and integrity were tested, it was randomly sheared by Covaris ultrasonic disruptor. Illumina sequencing pair-end libraries with an insert size of 300 bp were prepared using Nextera DNA Flex Library Prep Kit (Illumina, San Diego, CA, USA). Sequencing was performed using the Illumina NovaSeq platform (Illumina, San Diego, CA, USA). Raw reads were cleaned to discard low-quality reads (reads with adaptors or unknown nucleotides (Ns) or reads with more than 20% low-quality bases) using the SOAPnuke (v2.1.4) tool (https://github.com/BGI-flexlab/SOAPnuke) and, after data filtering, clean data were used for subsequent analyses.

For Oxford Nanopore sequencing, the libraries were prepared using the SQK-LSK109 ligation kit and using the standard protocol. The purified library was loaded onto primed R9.4 Spot-On Flow Cells and sequenced using a PromethION sequencer (Oxford Nanopore Technologies, Oxford, UK) with 48-h runs at Wuhan Benagen Tech Solutions Company Limited, Wuhan, China. Base-calling analysis of raw data was performed using the Oxford Nanopore GUPPY software (v0.3.0).

### RNA library construction, sequencing and data processing

For gene prediction analysis, total RNA was extracted from young leaves of *T. yunnanensis* using the RNA prep Pure Plant Plus Kit according to the manufacturer’s instructions (Tiangen Biotech (Beijing) Co., Ltd., China). RNA samples were pooled and used a strand-switching method and the cDNA-PCR Sequencing Kit (SQK-PCS109) to carry out sequencing of cDNA by PromethION sequencer (Oxford Nanopore Technologies, Oxford, UK).

To analyze the gene expression pattern, total RNA was extracted from 15 samples that comprise three biological replicates of independent samples of five tissues (root, stem, twig bark, bark and leaf) and sequencing was performed using the Illumina NovaSeq platform (Illumina, San Diego, CA, USA). The stem refers to the young shoots ca. 1 mm in diameter; twig bark is the bark of lateral branch about 0.5 cm in diameter; bark refers to the bark of the tree trunk about 5 cm in diameter.

RNA-seq reads were mapped to the reference genome assembly using STAR (v2.5.1b; parameters: -two pass Mode)^30^ and the FPKM was calculated to evaluate the expression level of each gene using the HTSeq (v.0.11.2) tool^31^ after averaging some replicated samples. DESeq was used for normalizing gene expression (Base Mean) in each sample, and for identifying differentially expressed genes (DEGs) for each compared group by using *P*-adj (adjusted *P* value) < 0.05 as the threshold. Co-expression network analysis (Pearson correlation coefficient > 0.8, *P* < 0.05) was performed and the co-expressed genes that may share taxol metabolism were characterized.

### Genome assembly

Based on the sequencing data of *T. yunnanensis*, the K-mer analysis method was used to estimate the genome size and heterozygosity using the kmer_freq program in the GCEpackage (v.1.0.0).

Genomic assembly was performed using SMARTdenovo software (https://github.com/ruanjue/smartdenovo). Two rounds of error correction were performed on the assembly result based on the nanopore sequencing data using Racon (v1.4.11) (https://github.com/isovic/racon). Two rounds of error correction were performed on the assembly result based on the Illumina Novaseq sequencing data using Pilon (v1.23)^32^. Finally, the genome was removed from the heterozygous sequences using the Purge_haplotigs pipeline (v.1.0.4)^33^ to obtain the final assembly result. To evaluate the genome, BUSCO (v.4.1.2)^18^ assessments and the Illumina short reads were aligned to the assembled genome using BWA, resulting in a mapping rate of 99.64%.

### Hi-C sequencing and data processing

High-quality DNA extracted from young leaves of *T. yunnanensis* was used for Hi-C sequencing. Formaldehyde was used for fixing chromatin. In situ Hi-C chromosome conformation capture was performed according to the DNase-based protocol described by Ramani^34^. The libraries were sequenced using 150 bp paired-end mode on an Illumina NovaSeq (Illumina, San Diego, CA, USA). For pseudochromosome level scaffolding, we used the assembly software ALLHIC (v0.9.12) for stitching, and then we imported the final files (.hic and .assembly) generated by the software into Juicebox (v1.11.08)^35^ for manual optimization.

### Repeat sequence annotation

We identified de novo repetitive sequences in the *T. yunnanensis* genome using the RepeatModeler (v.1.0.4) (https://github.com/rmhubley/RepeatModeler) software. After combining known repetitive sequences of RepeatMasker^36^ library and the de novo repetitive sequences constructed by RepeatModeler, we used RepeatMasker (v.4.0.5)^36^ (http://www.repeatmasker.org/) for genome repeat annotation. We used Genometools (v1.5.9)^37^ (-motif tgca -motifmis 1 -minlenltr 100 -maxlenltr 3000 -mintsd 4 -maxtsd 20) to detect full-length LTR retrotransposons in four gymnosperms and three angiosperm (*T. yunnanensis*, *G. montanum*, *G. biloba*, *S. giganteum*, *A. trichopoda*, *O. sativa* and *A. thaliana*) genomes. Further, we used tBLASTn (V2.2.26) to identify Copia and Gypsy super-families in seven genomes based on the reported Gypsy and Copia reverse transcriptase domains with sequences EAYLDDLASRSRKRKDHPTHLRLIFERoCRYFRIRLNPNKCSFCVTSGRLLGFIVSTTGIMVDPLKVGAIVQLPPPRTIVQLQSLQGKANFLRRFIANYAE and WKVYQMDVKSAFLNGYLEEEVYVQQPPRYEVRGQEDKVYRLKKALNGLKQAPRAWYSKIDSYMIKNEFIRSTSEPTLYTKVNEQGQILIVCLYVDDLIY, respectively^16^. Target hits were obtained using a strict filter criteria of identity ≥50% (Gypsy) and 60% (Copia) and coverage ratio ≥0.90. Then, the resultant amino acid sequences were aligned using MUSCLE (V3.8.31)^38^ with default parameters. Phylogenetic trees were inferred based on multiple sequence alignment using FastTree (V2.1.9)^39^. The integration times (*t*) of intact LTRs were estimated using the equation *t* = *K*/2*r*, where *K* is the number of nucleotide substitutions per site between each LTR pair and *r* is the nucleotide substitution rate, which was set to 7.34573 × 10^−10^ substitutions per site per year^11^.

### Gene prediction

Evidence from transcript mapping, ab initio gene prediction, and homologous gene alignment was combined to predict protein-coding genes in the *T. yunnanensis* genome. ONT cDNA reads from *T. yunnanensis* were aligned against the genome using Minimap2 (v2.17)^40^. Transcripts were assembled using stringtie2 (v2.1.5)^41^ and all assembled transcripts ORF were predicted by TransDecoder (v5.1.0) (https://github.com/TransDecoder/TransDecoder). Augustus (v3.3.2)^42^, Genscan (v1.0) (http://bioinf.uni-greifswald.de/webaugustus/predictiontutorial) and GlimmerHMM (v3.0.4) (http://ccb.jhu.edu/software/glimmer/index.shtml) were used for ab initio gene prediction. For homologous gene alignment, the proteins from four relative species (*Picea abies, Pinus lambertiana, Pinus taeda* and *S. giganteum*) were aligned to the genome using Exonerate (v2.4.0) (https://github.com/nathanweeks/exonerate). Finally, Use MAKER (v2.31.10) software (http://yandell.topaz.genetics.utah.edu/cgi-bin/maker_license.cgi) to integrate gene sets predicted by three methods and remove incomplete genes and genes with too short CDS (CDS length < 150 bp), a non-redundant and more complete gene set were obtained. We employed the BUSCO software (v.4.1.2)^18^ for evaluating the quality of the prediction based on the eukaryotic and embryophyta database.

### Gene function annotation

Functional annotation of the predicted protein-coding genes was carried out by performing Blastp (*e* value cut-off 1e−05) searches against entries in both the NCBI nr and Uniprot databases (http://www.uniprot.org/). Searches for gene motifs and domains were performed using InterProScan (v5.33)^43^ and HMMER (v3.1). The GO terms (http://geneontology.org/) for genes were obtained from the corresponding InterPro (https://github.com/ebi-pf-team/interproscan) or Uniprot entry (https://www.uniprot.org/). Pathway annotation was performed using KOBAS (v3.0) (https://github.com/xmao/kobas) against the KEGG database.

### Phylogenetic tree construction

All amino acid sequences of the 14 selected species were aligned using Blastp (v.2.6.0; parameter: -evalue 1e−5 -outfmt 6), and the gene family clustering was performed using OrthoMCL software (v.2.0.9; parameters: percentMatchCutoff = 30, evalueExponentCutoff = 1e−5, expansion coefficient 1.5)^44^.

A single-copy gene family shared by at least eight selected species (575 gene families) was screened to construct phylogenetic trees. The 575 gene family files were each aligned using MUSCLE (v.3.8.31)^38^, both as amino acid and nucleotide, resulting in two distinct alignments per gene family. We also forced nucleotide sequences on the amino acid alignments using a custom Perl script to obtain codon-preserving alignments of nucleotide sequences. Gene trees were then reconstructed for each gene family using RAxML (v.8.2.10) software^45^ with 100 replicates of bootstrapping. For each gene family, we estimated four different gene trees based on: amino acid alignments, DNA alignment, codon alignment (nucleotides forced to the amino acid alignment), and codon 1 and 2 alignment (codon alignments where the third codon position was removed). Nucleotide-based analyses were conducted using the GTR + GAMMA model; for amino acid analyses, we used WAG model. For four different datasets, we inferred four maximum likelihood tree of species from gene trees using RAxML (v.8.2.10) software^45^. A phylogenetic tree of each single-copy gene was further constructed to infer a consensus species tree using ASTRAL (v5.7.1)^46^.

For supermatrix analyses, we concatenated all gene alignments and ML supermatrix analyses were performed using RAxML (v.8.2.10)^45^ software. In this study, four supermatrix datasets were created for amino acid, codon alignment (nucleotides forced to the amino acid alignment), nucleotide alignments and codon1, 2 alignment (codon alignments where the third codon position was removed). These data matrices were used for maximum likelihood phylogenetic analyses by RAxML (v.8.2.10)^45^ with the GTR + GAMMA models for nucleotide and WAG models for amino acid data. For each analysis, support was inferred for branches on the final tree from 100 bootstrap replicates.

Based on the phylogenetic tree result, the mcmctree of PAML (v.4.9; parameter: nsample = 1000000; burnin = 200000; seqtype = 0; clock = 3; model = 4)^47^ was used to estimate the divergence time of the different species. Published divergence times^48^ for *A. thaliana* and *V. vinifera*: 90−120 MYA, *N. colorata* and *A. trichopoda*: 215–265 MYA, *P. abies* and *P. taeda*: 123–220 MYA, *S. moellendorffii* and *P. abies*: 410–440 MYA, *P. abies* and *A. trichopoda*: 250–390 MYA and *O. sativa* and *V. vinifera*: 125−150 MYA were used to calibrate the divergence time.

Gene family contraction and expansion analysis were performed using CAFÉ (v.2.1; parameter:–filter) software^49^ based on gene family clustering results.

### Whole-gene duplication analysis

All *T. yunnanensis* amino acid sequences were self-aligned using Blastp (*e* value cut-off 1e−05) and the best Blastp result was retained. To obtain paralogous gene families, we performed gene cluster analyses based on the CDS alignment using OrthoMCL (v 2.0.9)^44^. *Ks* values were calculated from all paralogous families using yn00 in the PAML package^47^. The *Ks* of a given family was represented by the median value, and the distribution of corrected *Ks* values was plotted by median values^16^.

To distinguish whether this peak represents a whole-genome duplication event or background small-scale duplications, we identified paralogous gene pairs using Blastp methods and determined syntenic blocks using MCScanX^50^ (https://github.com/wyp1125/MCScanx). Although the synonymous substitution rate (*Ks*) was calculated for *T. yunnanensis* syntenic block gene pairs and *Ks* distribution clearly showed a major peak at around 0.1, there were no widespread and well-maintained one-versus-one syntenic blocks indicates that a recent whole-genome duplication (WGD) event has not occurred in the *T. yunnanensis* genome. Indeed, analysis of duplication types of the *T. yunnanensis* paralogs by Duplicate_gene_classifier tool of MCScanX^50^ indicates that there are four types: WGD/segmental duplication (match genes in syntenic blocks), dispersed (other modes than segmental, tandem and proximal), proximal (in the nearby chromosomal region but not adjacent) and tandem (continuous repeat).

### Identification of genes related to the Taxol pathway in *T. yunnanensis*

The genes related to the Taxol pathway in *T. yunnanensis* were obtained by BLAST based on the reported Taxol pathway genes (GGPPS, TXS, T5αOH, T10βOH, T2αOH, T7βOH, T13αOH, TAT, DBAT, TBT, BAPT and DBTNBT) (the reference gene accession number in Supplementary Data 8). Thirty-eight genes were identified based on Sequence identity with reference genes.

The sequences of the *A. thaliana* and rice CYP genes were downloaded (http://drnelson.uthsc.edu/P450seqs.dbs.html) and used as queries to search for homologs and conserved domains (PF00067) against the *T. yunnanensis* genome. The classification of TyunCYP450 proteins was based on reference sequences from a P450 database established by Nelson^51^.

All of the hydroxylation-like genes responsible for C-2, C-5, C-7, C-10, C-13 and C-14 hydroxylation in Taxol pathway belonged to the CYP725 sub-family. The CYP725 genes were identified in *T. yunnanensis*, *S. giganteum*, *P. menziesii* and *G. biloba* genome. The CYP725 phylogenetic trees were constructed using the maximum likelihood (ML) method. MAFFT (v7.397)^52^ was used for multiple sequence alignments (–maxiterate 1000 –localpair), and RAxML (v8.2.10)^45^ was used for tree building with bootstrapping set to 1000.

### Genome mining for gene clusters of the Taxol pathway in *T. yunnanensis*

To search for potential gene clusters that are associated with the Taxol pathway in *T. yunnanensis*, the genes CYP725A genes and genes related to the Taxol pathway with a distance <10 apart, are considered to be a gene cluster. The gene distance represents the number of genes between two focal genes. The results were parsed and summarized with additional Pfam (version 31.0) entries and gene expression patterns across five tissue types (Supplementary Data 10).

### Statistics and reproducibility

Fifteen samples comprise three biological replicates of independent samples of five tissues for RNAseq analysis. All statistical tests were performed by publicly available programs and packages as described in the ‘Methods’ section. Reproducibility can be accomplished by the sample collection and laboratory methods described in the ‘Methods’ section and all the data of our analysis availed in public databases.

### Reporting summary

Further information on research design is available in the Nature Research Reporting Summary linked to this article.

## Supplementary information

Supplementary information

Description of Additional Supplementary Files

Supplementary Data

Reporting Summary

## Data Availability

The data supporting the findings of this work are available within the paper and its Supplementary Information files. The datasets generated and analyzed during this study are available from the corresponding authors upon request. The genome sequence data and the transcriptome sequence data for *T. yunnanensis* have been deposited under NCBI BioProject number PRJNA661543.
