# Supplementary material for: Taxus yunnanensis genome offers insights into gymnosperm phylogeny and taxol production
Source: Commun Biol. 2021 Oct 20;4:1203. doi: 10.1038/s42003-021-02697-8 (PMC8528922; doi:10.1038/s42003-021-02697-8)
Supplement: Supplementary file 1 — Supplementary information [file 42003_2021_2697_MOESM1_ESM.pdf]

Supplementary information

Supplementary Figures

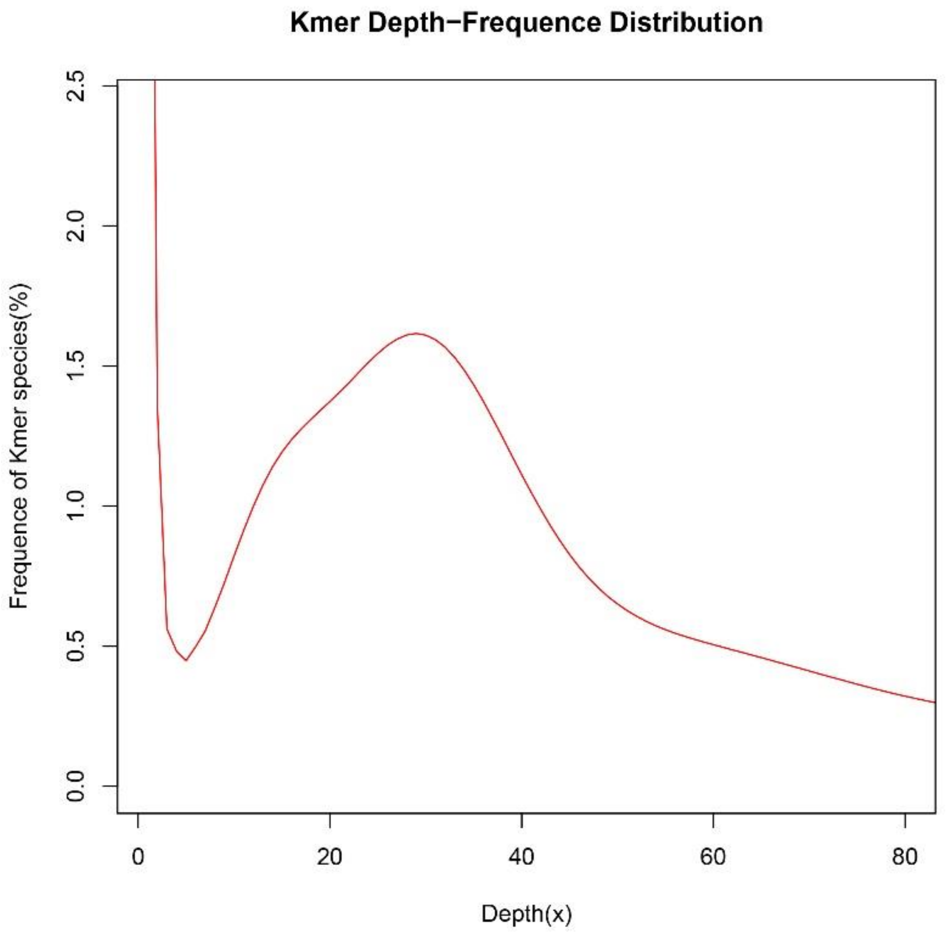

Supplementary Figure 1 17-mer frequency distribution of *Taxus yunnanensis* genome sequencing

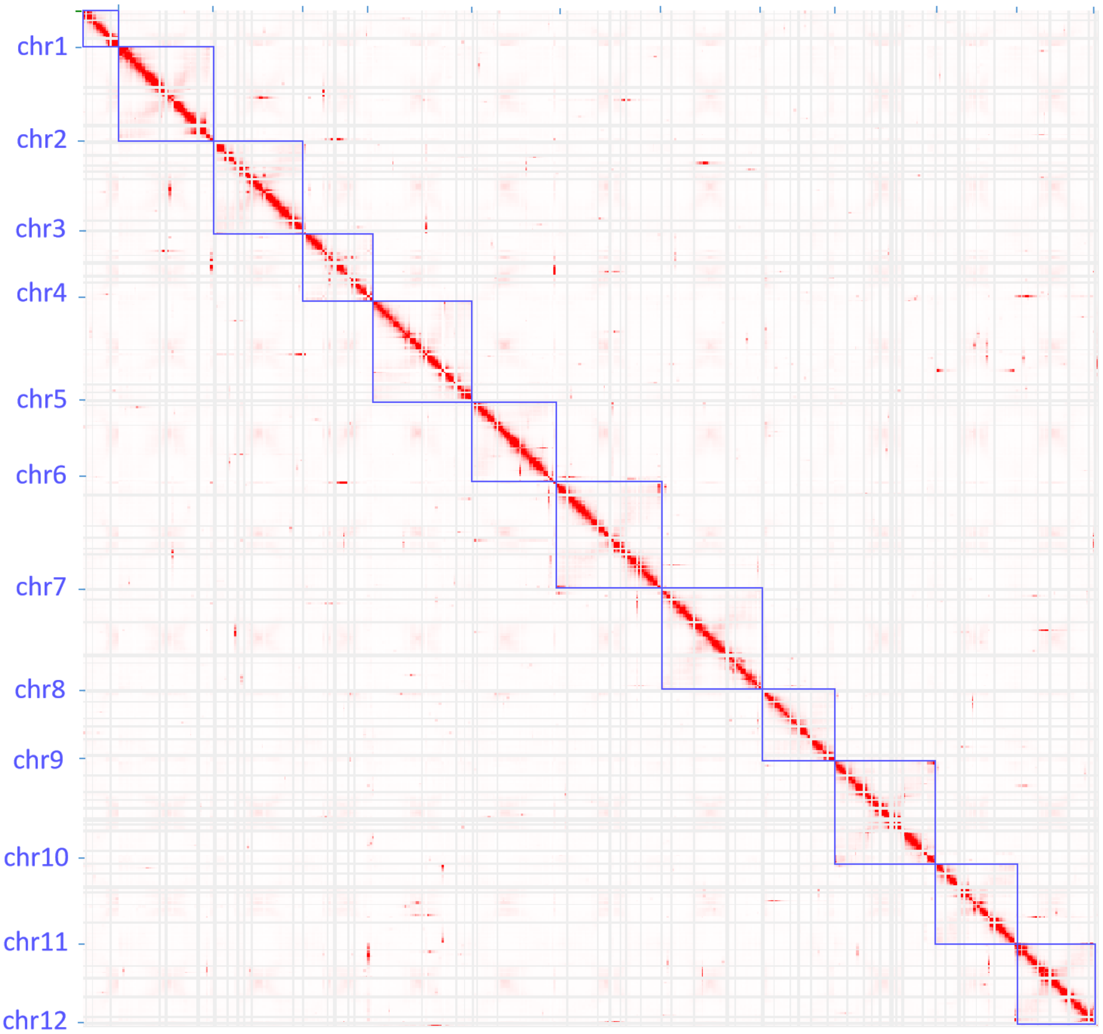

Supplementary Figure 2 The interrelationships between chromosomes of *T. yunnanensis*.

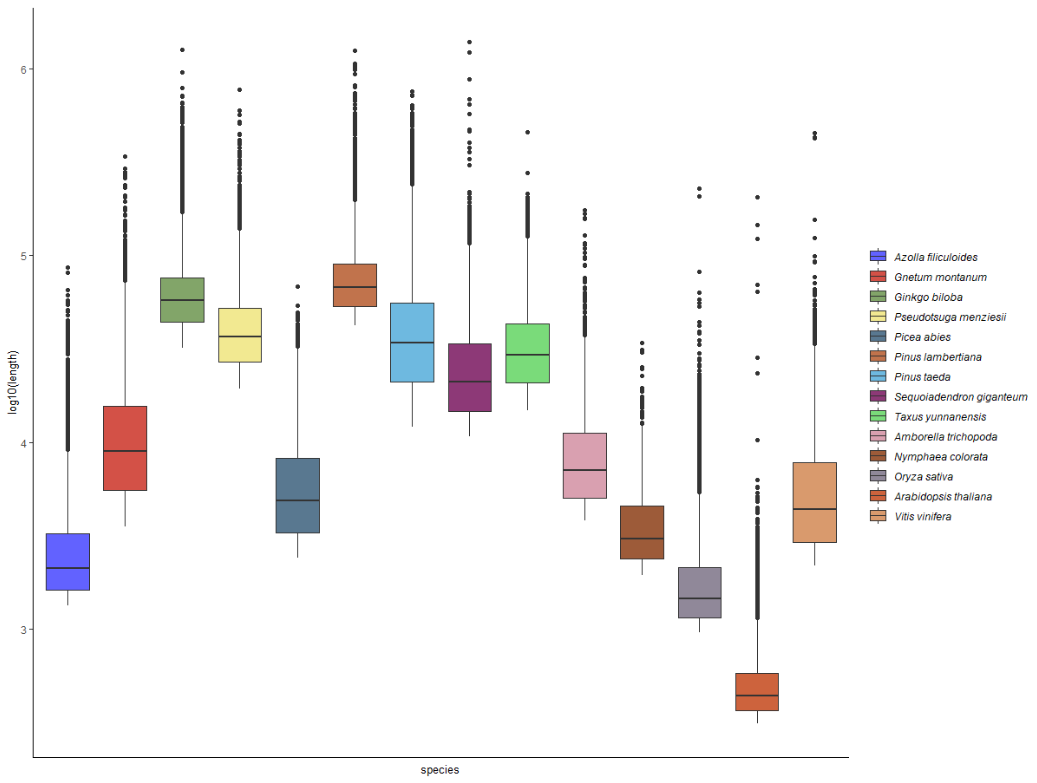

Supplementary Figure 3 Comparison of the longest 10% of introns in the 14 land plants. Box edges represent the 0.25 quantile and 0.75 quantile with the median values shown by bold lines. Whiskers extend to data no more than 1.5 times the interquartile range, and remaining data are indicated by dots.

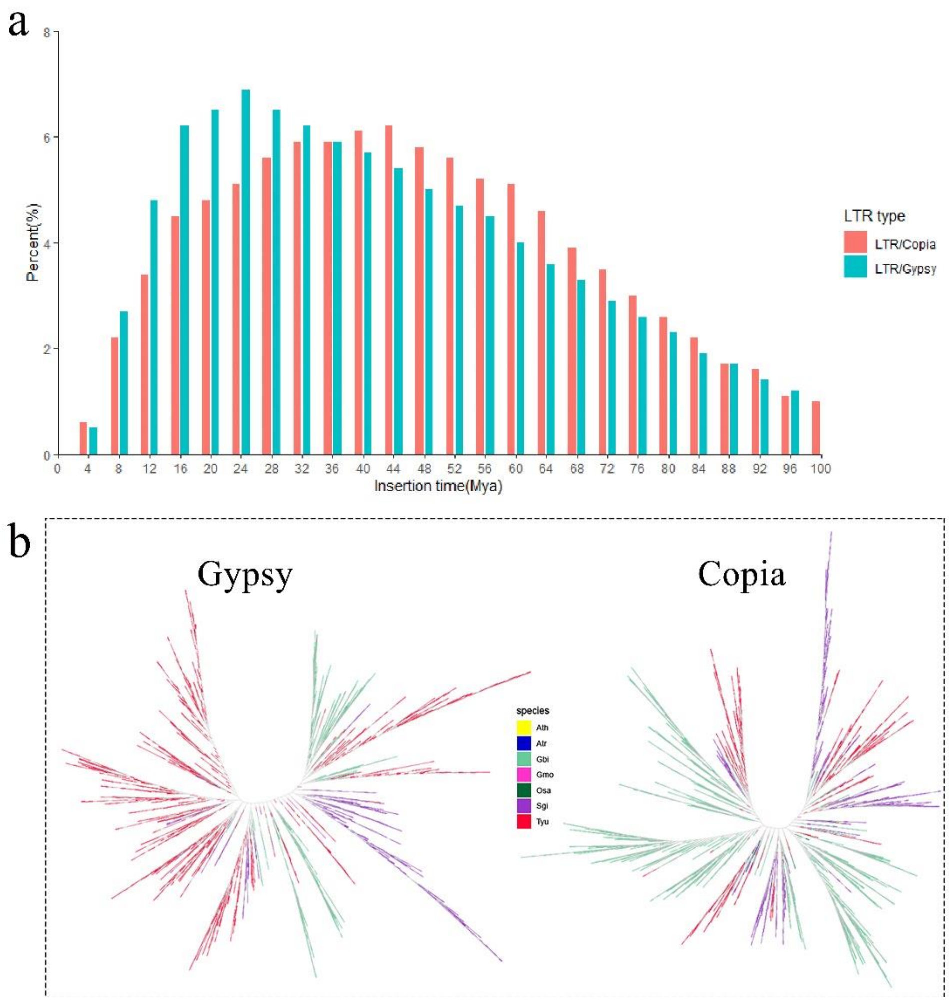

Supplementary Figure 4 Evolution of LTR-RTs in *T. yunnanensis*. (a) The timing of LTR-RTs (Gypsy and Copia) insertions of *T. yunnanensis* based on a mutation rate of 7.34573×10^{-10} substitutions per base per year. (b) A phylogenetic relationship within the LTR-RTs in four gymnosperms and three angiosperms. Tyu, *T. yunnanensis*; Gmo, *G. montanum*; Gbi, *G. biloba*; Sgi, *S. giganteum*; Atr, *A. trichopoda*; Osa, *O. sativa*; Ath, *A. thaliana*.

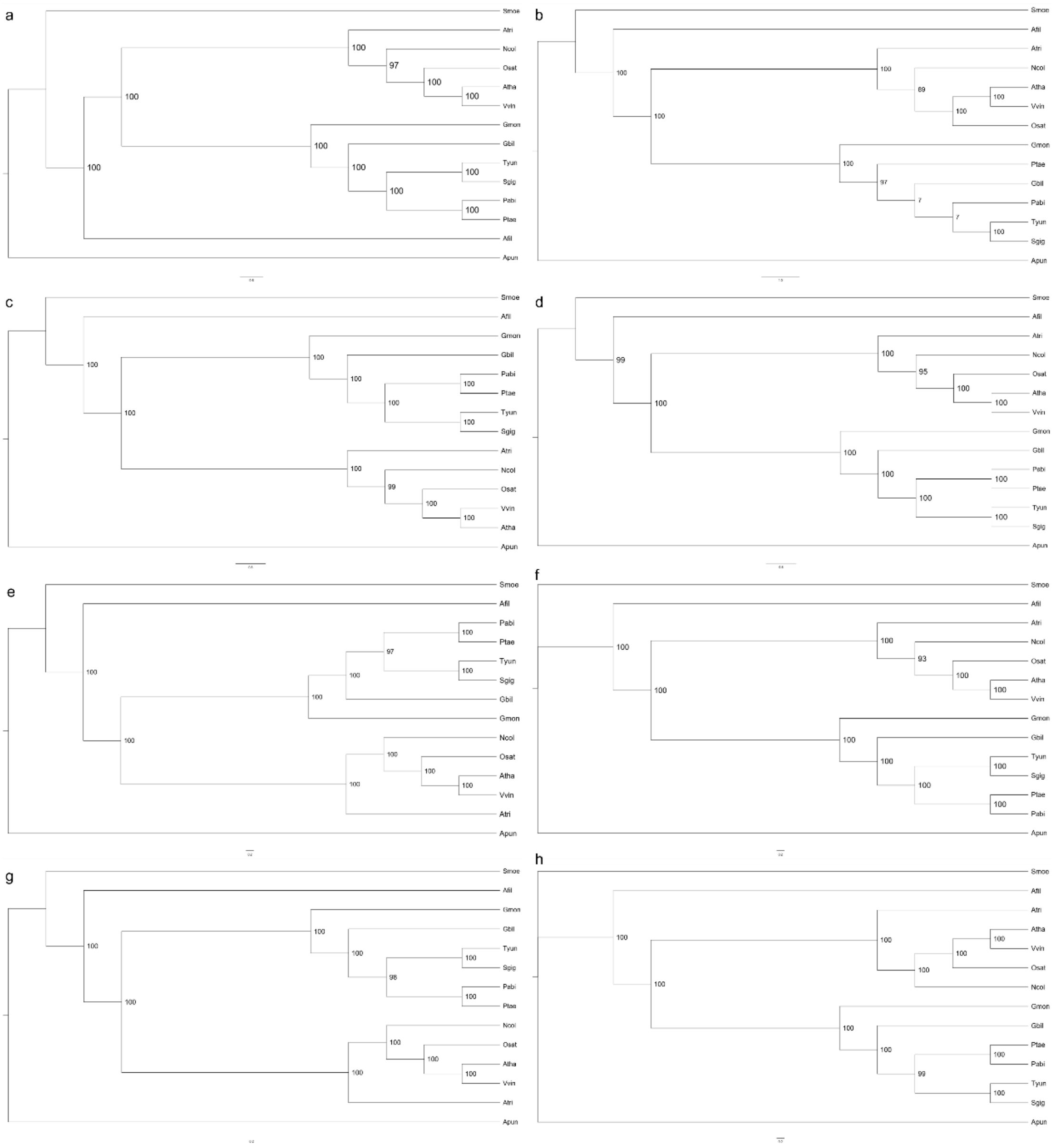

Supplementary Figure 5 Inferred phylogenetic tree of 14 plant species in. (a) ASTRAL tree based on amino acid alignments; (b) ASTRAL tree based on DNA alignment; (c) ASTRAL tree based on codon alignments; (d) ASTRAL tree based on codon alignments with third-position removed; (e) Supertree tree based on amino acid alignments; (f) Supertree tree based on DNA alignment; (g) Supertree tree based on codon alignments; (h) Supertree tree based on codon alignments with third-position removed.

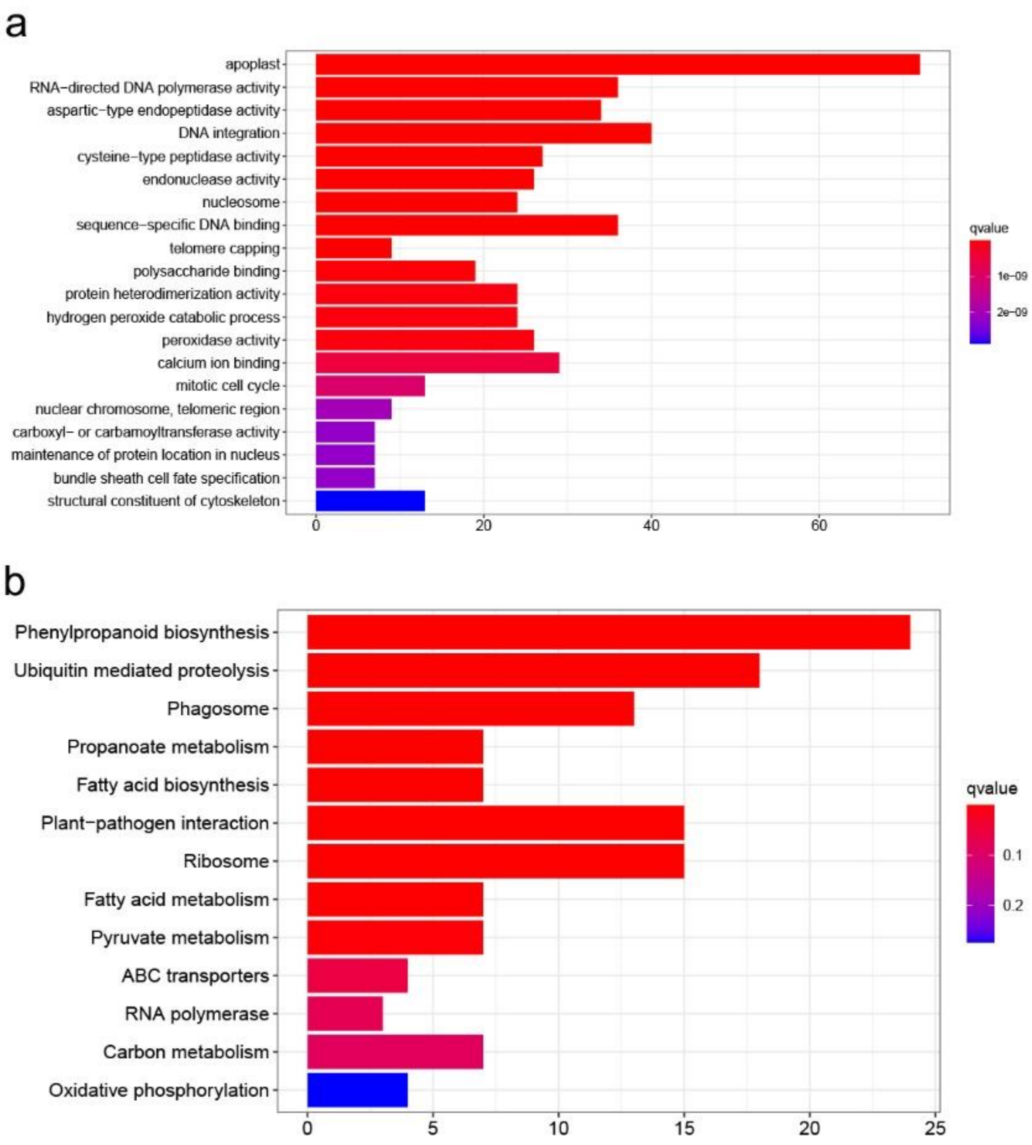

Supplementary Figure 6 (a) GO function enrichment of significantly expanded genes of *T. yunnanensis*. * $P < 0.05$ by hypergeometric test and FDR adjustments. (b) KEGG enrichment of expanded genes of *T. yunnanensis*. * $P < 0.05$ by hypergeometric test and FDR adjustments.

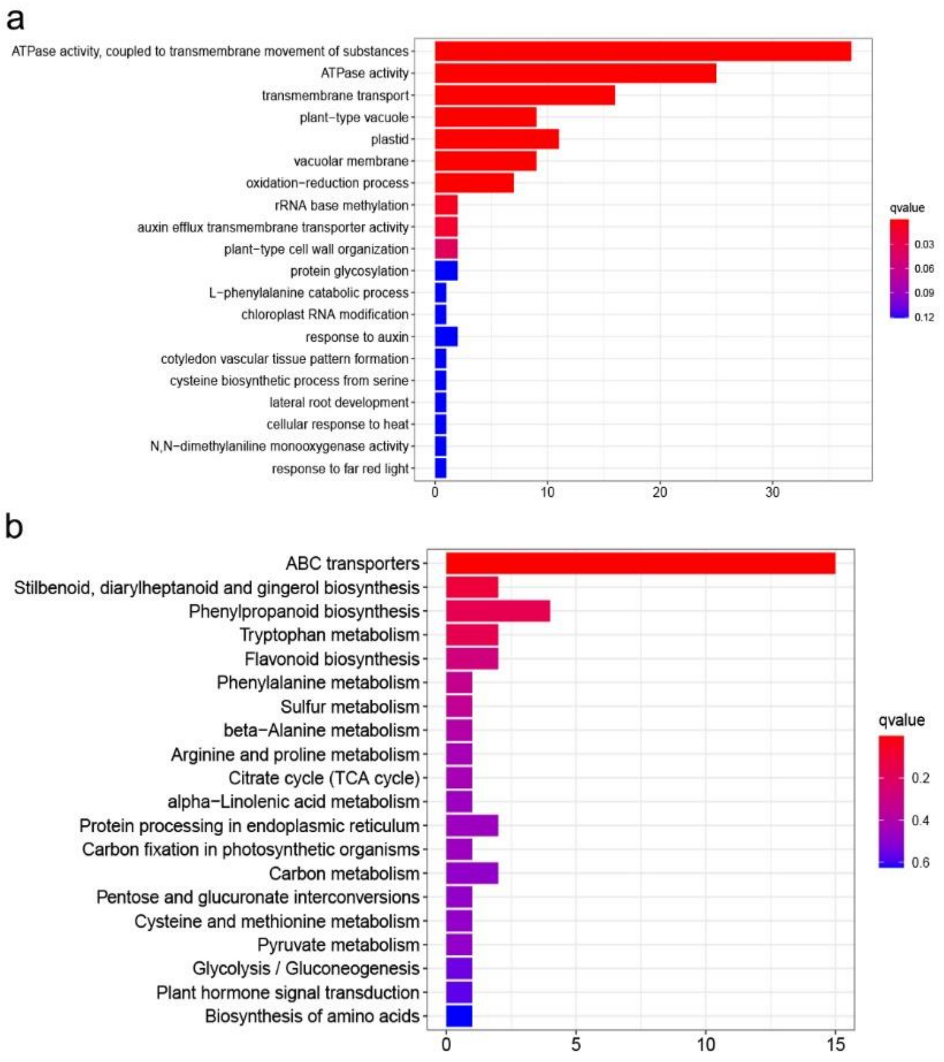

Supplementary Figure 7 (a) GO function enrichment of significantly contracted genes of *T. yunnanensis*. * $P < 0.05$ by hypergeometric test and FDR adjustments. (b) KEGG enrichment of contracted genes of *T. yunnanensis*. * $P < 0.05$ by hypergeometric test and FDR adjustments.

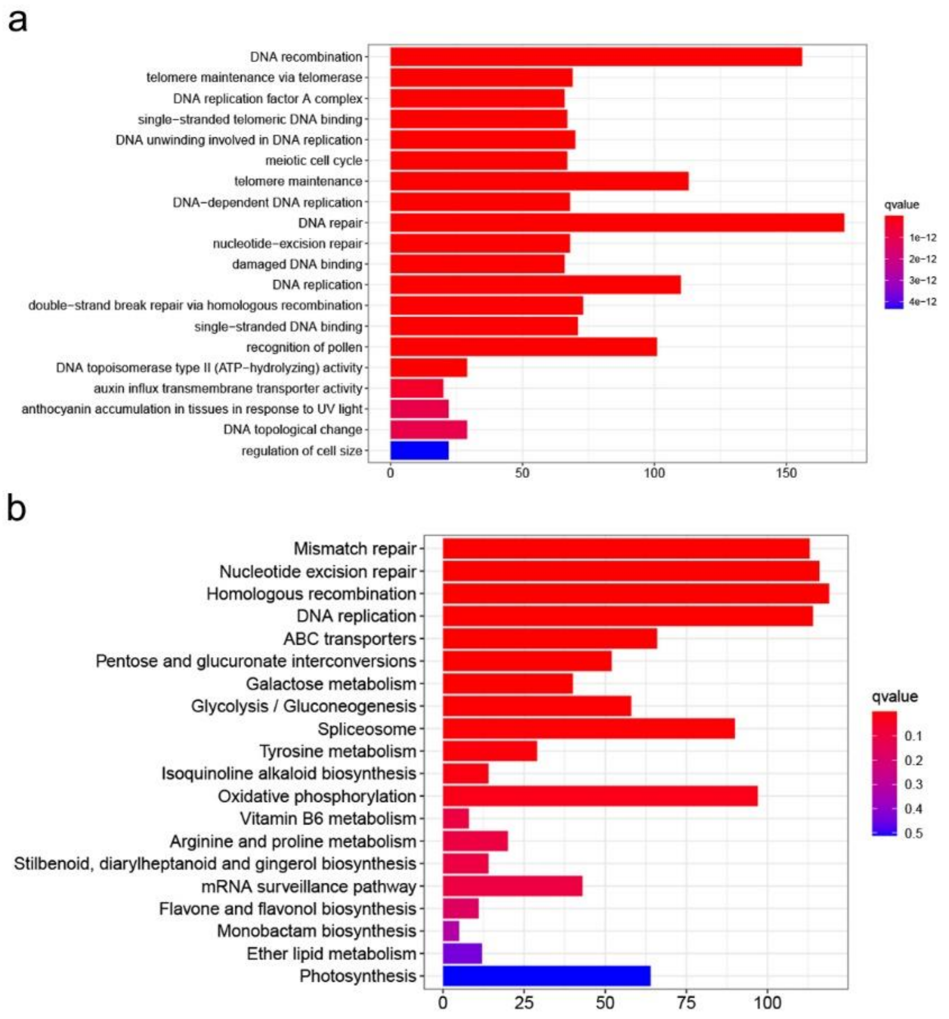

Supplementary Figure 8 (a) GO function enrichment of unique genes of *T. yunnanensis*. * $P < 0.05$ by hypergeometric test and FDR adjustments. (b) KEGG enrichment of unique genes of *T. yunnanensis*. * $P < 0.05$ by hypergeometric test and FDR adjustments.

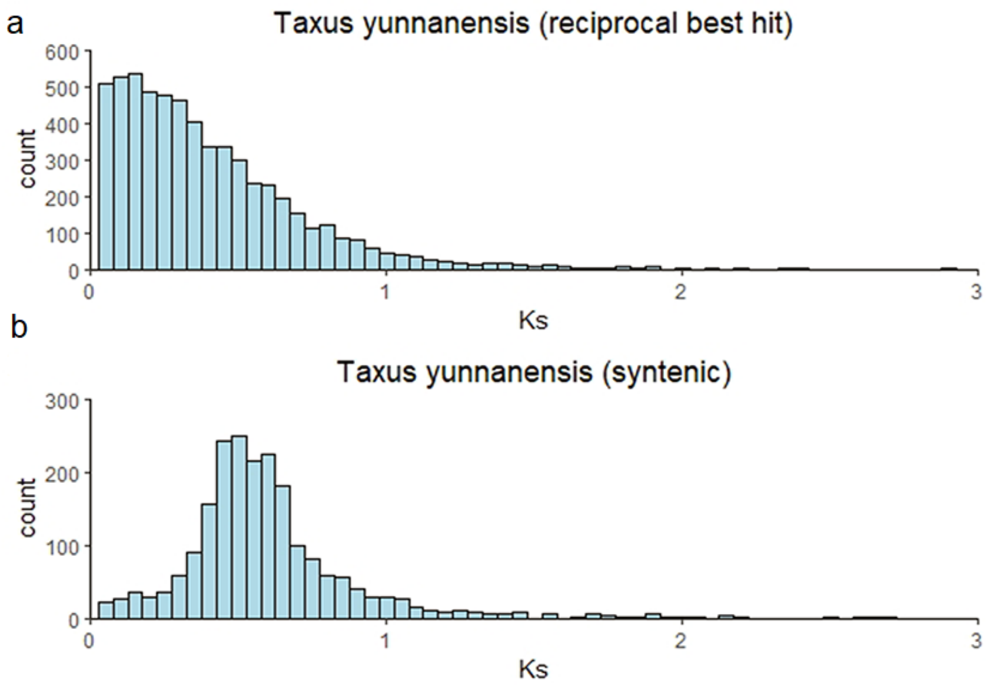

Supplementary Figure 9 Histogram distribution of *K*s (synonymous substitution rate) for paralogous gene pairs identified through (a) reciprocal best hit analysis (ABCGHI) and (b) syntenic block gene pairs identified through MCScanX (DEFJKL) in *T. yunnanensis*.

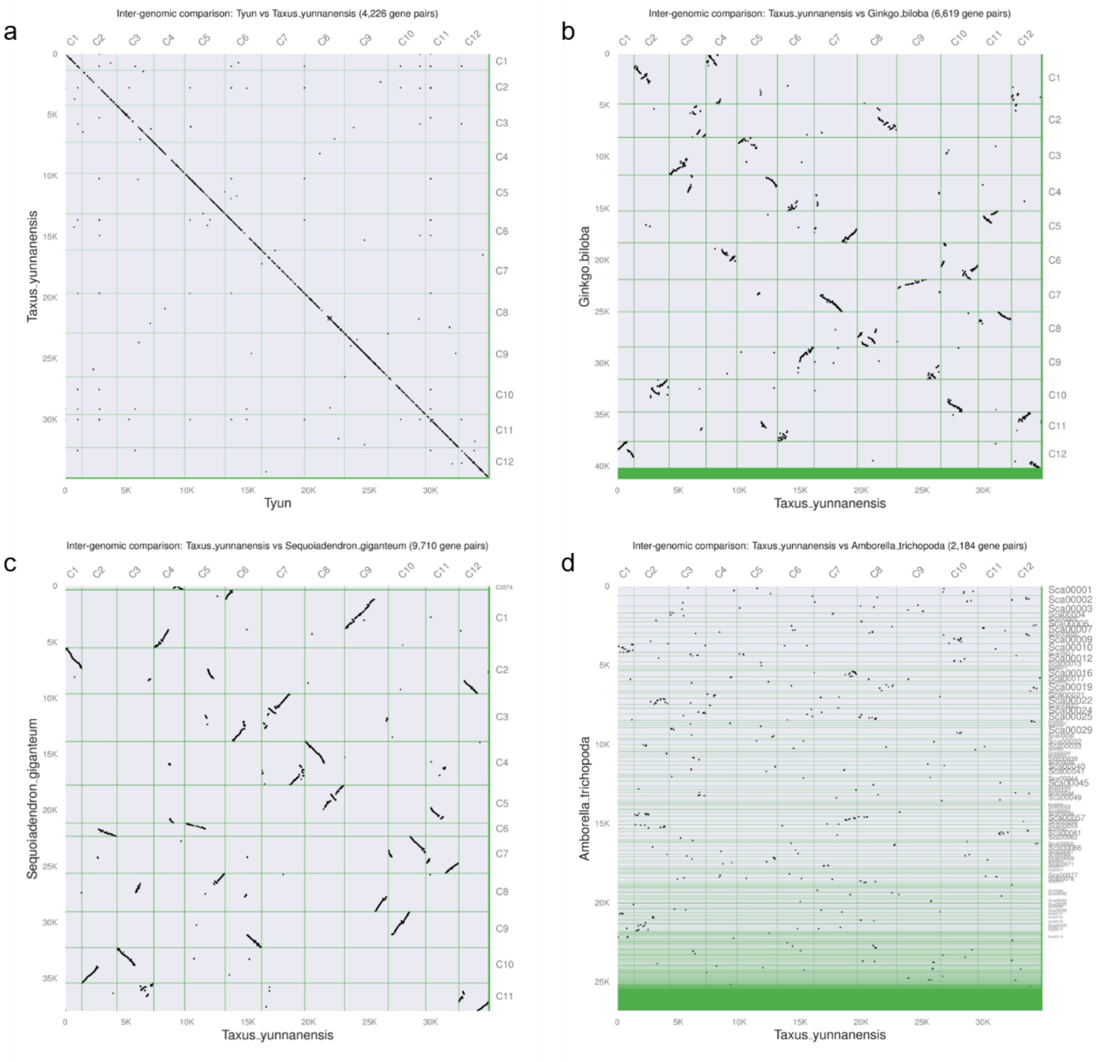

Supplementary Figure 10 Synteny analysis within the *T. yunnanensis* genome and between the *T. yunnanensis* and other plant genomes. (a) Dot plot of paralogs in *T. yunnanensis*; (b) Dot plot illustrating the comparative analysis of the *T. yunnanensis* and *G. biloba*; (c) Dot plot illustrating the comparative analysis of the *T. yunnanensis* and *S. giganteum*; (d) Dot plot illustrating the comparative analysis of the *T. yunnanensis* and *A. trichopoda*

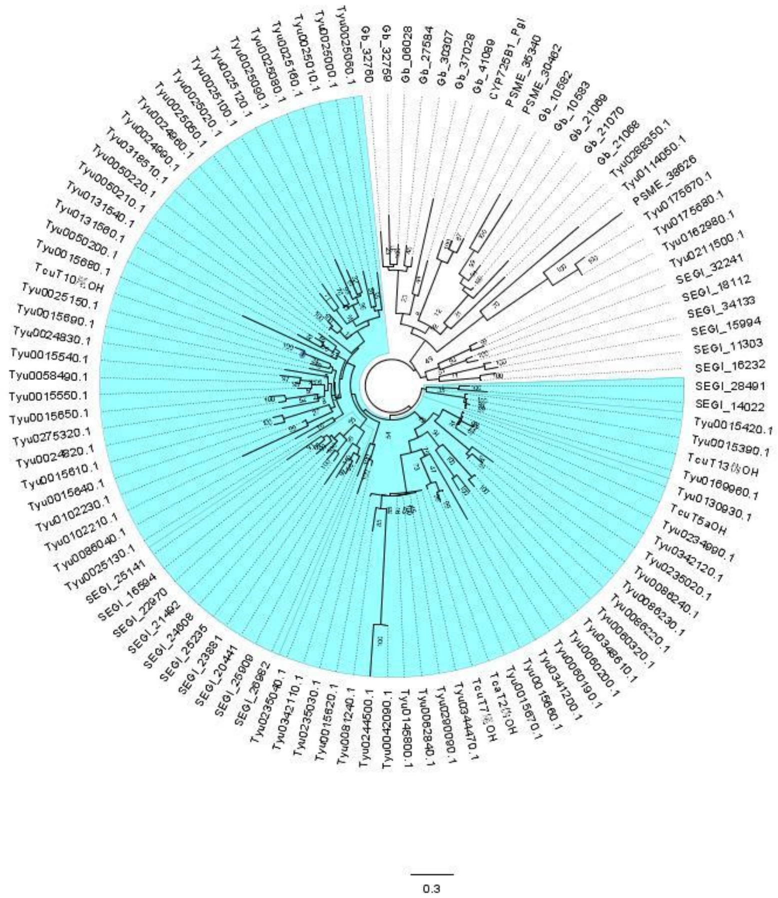

Supplementary Figure 11 Phylogenetic tree of the CYP725 gene subfamily in *T. yunnanensis*, *S. giganteum*, *P. menziesii* and *G. biloba*. Genes of the CYP725A gene subfamily are labeled in blue.

Supplementary Tables

Supplementary Table 1 Survey statistic results of *T. yunnanensis*

Sample	K-mer	K-mer Number	K-mer Depth	Genome Size（G）	Heterozygous Ratio（%）	Repeat（%）
T. yunnanensis	17	301,172,711,110	28.71	10.49	0.54	77.74

Supplementary Table 2. Sequencing data statistics of *T. yunnanensis*

Oxford Nanopore sequencing data								
**Rank**	**Type**	**Total Base**	**Total Reads**	**Max Len**	**Avg Len**	**N50**	**N90**	**meanQ**
>0	all	857,454,433,180	39,648,721	2,614,001	21,626	32,397	13,435	8.42
>0	pass	740,286,432,630	31,080,271	338,250	23,819	33,019	13,807	9.34
>0	fail	117,168,000,550	8,568,450	2,614,001	13,674	28,518	10,965	5.09
>1000	all	855,763,759,169	36,088,429	2,614,001	23,713	32,456	13,504	8.68
>1000	pass	739,713,498,232	29,932,573	338,250	24,713	33,043	13,834	9.36
>1000	fail	116,050,260,937	6,155,856	2,614,001	18,852	28,789	11,393	5.4
>2000	all	854,244,328,547	35,054,732	2,614,001	24,369	32,509	13,565	8.72
>2000	pass	738,876,152,997	29,369,435	338,250	25,158	33,076	13,873	9.36
>2000	fail	115,368,175,550	5,685,297	2,614,001	20,292	28,953	11,631	5.45
>5000	all	845,751,298,257	32,594,489	2,614,001	25,948	32,804	13,906	8.79
>5000	pass	733,203,737,989	27,754,235	338,250	26,418	33,307	14,139	9.37
>5000	fail	112,547,560,268	4,840,254	2,614,001	23,252	29,641	12,509	5.49
>10000	all	824,543,453,717	29,645,916	2,614,001	27,813	33,551	14,751	8.84
>10000	pass	717,296,040,317	25,537,309	338,250	28,088	33,956	14,878	9.38
>10000	fail	107,247,413,400	4,108,607	2,614,001	26,103	30,963	13,996	5.53
**Illumina NovaSeq platform sequencing data**								
**Sample**	**Total Base (G)**	**Clean Base(G)**	**Q20(%)**	**Q30(%)**	**GC Content(%)**			
T. yunnanensis	500	495.46	98.28	92.74	37.26			
**HIC**								
**Sample**	**Total Base**	**Read Length**	**Q20(%)**	**Q30(%)**	**GC Content(%)**			
T. yunnanensis	601,553,339,400	150	97.4	90.9	36.7			

Supplementary Table 3. Chromosome data statistics of *T. yunnanensis*

Chromosome	Length (bp)	Contig_Num
chr1	421,506,113	387
chr2	1,038,231,987	773
chr3	966,801,426	835
chr4	702,045,394	803
chr5	1,031,922,046	1420
chr6	880,577,571	930
chr7	1,053,173,319	1064
chr8	1,071,627,631	1179
chr9	761,943,471	1205
chr10	1,040,677,373	893
chr11	854,835,189	1054
chr12	801,891,208	599
chr0	113,083,356	138

Supplementary Table 4. Completeness of assembly and gene sets assessed with BUSCOv4.1.2

			Genome assembly BUCSO		Gene sets BUSCO	
Species	Genome size (Gb)	Gene number	Eukaryotes（255）	Embryophyta（1614）	Eukaryotes（255）	Embryophyta（1614）
Taxus yunnanensis	10.73	30,766	72.6%	52.5%	73.7%	52%
Gnetum montanum	4.07	27,491	91.0%	74.4%	96.1%	83.8%
Ginkgo biloba	10.61	41,840	52.5%	45.3%	71.4%	60%
Pseudotsuga menziesii	15.7	51,419	90.5%	52.1%	90.6%	68.5%
Picea abies	19.6	70,736	35.7%	32.0%	36.9%	28.1%
Pinus lambertiana	31	38,518	44.3%	36.9%	77.6%	73.3%
Pinus taeda	20.1	51,751	55.3%	39.7%	61.6%	41.7%
Sequoiadendron giganteum	8.13	37,936	78.5%	38.40%	64.4%	50%

Supplementary Table 5. Summary of the intron length in 14 land plants

Species	Max	Num	Mean	top10_max	top10_min	top10_Num	top10_mean
Azolla filiculoides	86,370	107,674	584.96	86,370	1,331	10,768	3,043.49
Gnetum montanum	342,127	95,009	1,769.04	342,127	3,536	9,501	13,322.20
Ginkgo biloba	1,272,918	136,069	7,883.69	1,272,918	31,990	13,607	65,777.14
Pseudotsuga menziesii	778,429	155,286	5,564.45	778,429	19,315	15,529	43,867.92
Picea abies	68,269	107,313	998.94	68,269	2,391	10,732	6,813.99
Pinus lambertiana	1,254,690	140,935	11,467.81	1,254,690	42,173	14,094	81,103.24
Pinus taeda	758,522	159,274	5,596.18	758,522	12,126	15,928	49337.87
Sequoiadendron giganteum	1,399,110	127,131	3,894.35	1,399,110	10,743	12,714	28,881.14
Taxus yunnanensis	462,177	96,238	5,002.89	462,177	14,790	9,624	35,282.16
Amborella trichopoda	175,748	82,937	1,527.54	175,748	3,785	8,294	9,342.10
Nymphaea colorata	33,902	142,571	755.627	33,902	1,931	14,258	3,772.19
Oryza sativa	228,480	115,917	437.36	228,480	956	11,592	2,181.66
Arabidopsis thaliana	206,015	113,846	164.06	206,015	311	11,385	591.99
Vitis vinifera	455,358	109,059	1,024.77	455,358	2,165	10,906	6,509.40

Note: Max, the length of the longest intron; Number, the number of the introns; Mean, the average length of the introns; Top 10, the length distribution for the 10% longest introns

Supplementary Table 6. Summary of Repeat contents in *T. yunnanensis* genome

Item	Subfamily	Number	Length(bp)	Coverage(%)
SINE	/	84	4,955	0.00%
LINE	/	258,718	193,031,889	1.80%
LTR	/	2,629,574	4,397,129,449	40.95%
LTR	Gypsy	2,138,065	3,860,557,122	35.95%
LTR	Copia	453,398	511,857,658	4.77%
DNA	/	382,779	252,186,558	2.35%
Satellite	/	82,043	47,354,435	0.44%
Simple_repeat	/	902,799	54,853,810	0.51%
Low_complexity	/	152,159	12,769,822	0.12%
Other	/	39,265	22,623,213	0.21%
Unknown	/	6,092,322	3,229,357,572	30.08%
Total	/	10,539,743	7,957,191,600	74.11%

Supplementary Table 7. Summary of LTR-RTs in four gymnosperms and three angiosperms

Species	LTR/Gypsy	LTR/Copia	Genome size
Taxus yunnanensis	173,798	42,161	10.7G
Gnetum montanum	64,727	2,480	4.07G
Ginkgo biloba	185,968	58,119	10.6G
Sequoiadendron giganteum	84,134	53,141	8.1G
Amborella trichopoda	1,674	1,057	700M
Oryza sativa	2,669	1,074	400M
Arabidopsis thaliana	359	239	120M

Supplementary Table 8. Summary of gene families in 14 plant genomes

Species	Genes number	Genes in families	Unclustered genes	Family number	Unique families	Average genes per family
Azolla filiculoides	20,203	15,083	5,120	9,219	762	1.64
Arabidopsis thaliana	27,416	22,455	4,961	12,668	1,029	1.77
Ginkgo biloba	41,309	26,487	14,822	13,684	1,547	1.94
Oryza sativa	55,803	41,820	13,983	13,412	2,455	3.12
Pinus taeda	51,751	40,671	11,080	16,579	3,149	2.45
Selaginella moellendorffii	34,799	32,043	2,756	12,166	3,999	2.63
Vitis vinifera	25,834	23,054	2,780	12,816	536	1.8
Anthoceros punctatus	25,431	18,583	6,848	8,836	978	2.1
Amborella trichopoda	26,846	18,158	8,688	12,162	1,007	1.49
Gnetum montanum	27,491	20,429	7,062	11,657	1,455	1.75
Nymphaea colorata	31,475	23,345	8,130	12,027	991	1.94
Picea abies	66,632	36,473	30,159	19,729	2,944	1.85
Sequoiadendron giganteum	37,936	28,064	9,872	14,339	1,329	1.96
Taxus yunnanensis	34,931	26,059	8,872	14,328	1,460	1.82

Supplementary Table 9. Summary of CYP genes in five species

Species	*Arabidopsis thaliana*	*Ginkgo biloba*	*Gnetum montanum*	*Picea glauca*	*Pseudotsuga menziesii*	*Sequoiadendron giganteum*	*Taxus yunnanensis*
CYP450	0	3	4	2	2	2	4
CYP51	1	1	1	1	4	2	6
CYP701	1	23	1	1	1	9	17
CYP702	6	6	0	0	0	0	0
CYP703	1	2	2	0	6	6	4
CYP704	3	11	18	4	27	11	7
CYP705	25	0	0	0	0	0	0
CYP706	8	1	0	0	0	1	2
CYP707	4	7	16	2	5	7	4
CYP708	4	4	0	0	0	0	0
CYP709	3	4	1	0	26	5	1
CYP71	50	0	8	0	1	0	3
CYP710	4	1	1	0	4	1	1
CYP711	1	2	1	1	11	2	1
CYP712	2	0	0	0	0	0	0
CYP714	2	0	0	0	0	0	0
CYP715	1	4	0	0	2	4	5
CYP716	3	16	13	3	15	59	31
CYP718	1	0	0	0	0	1	0
CYP72	9	0	1	0	1	0	0
CYP720	1	21	1	5	22	39	22
CYP721	1	0	1	0	0	0	0
CYP722	1	1	2	0	1	1	0
CYP724	1	1	1	1	3	2	1
**CYP725**	**0**	**12**	**0**	**1**	**3**	**19**	**68**
CYP727	0	2	1	1	2	2	1
CYP728	0	0	0	0	0	7	7
CYP729	0	7	0	0	0	8	5
CYP73	1	12	5	1	13	5	3
CYP733	0	0	0	0	0	3	0
CYP734	1	2	1	0	6	1	1
CYP735	2	4	2	0	15	10	3
CYP736	0	79	39	17	112	57	37
CYP74	2	8	10	5	19	5	9
CYP75	1	3	8	6	33	25	19
CYP750	0	16	55	6	163	94	80
CYP76	9	50	54	5	78	101	56

CYP77	5	8	4	1	17	10	22
CYP78	6	16	15	6	23	19	11
CYP79	10	0	0	0	0	20	2
CYP81	18	0	0	0	0	2	0
CYP82	5	6	0	0	0	30	32
CYP83	2	0	0	0	0	0	0
CYP84	2	0	1	0	0	0	0
CYP85	2	2	3	1	8	2	3
CYP86	11	35	8	18	47	32	56
CYP866	0	31	48	3	48	84	22
CYP867	0	2	0	1	22	5	6
CYP87	1	2	3	0	3	2	3
CYP88	2	2	2	1	1	1	1
CYP89	7	0	2	0	1	2	2
CYP90	4	5	8	3	13	12	5
CYP92	0	10	22	2	15	27	21
CYP93	1	1	0	0	0	4	1
CYP94	6	24	2	1	17	17	22
CYP96	13	0	1	0	0	0	0
CYP97	3	3	6	4	5	4	3
CYP98	3	4	1	1	2	4	14
	250	454	373	104	797	766	624
