# Supplementary material for: Taxus yunnanensis genome offers insights into gymnosperm phylogeny and taxol production
Source: Commun Biol. 2021 Oct 20;4:1203. doi: 10.1038/s42003-021-02697-8 (PMC8528922; doi:10.1038/s42003-021-02697-8)
Supplement: Supplementary file 2 — Description of Additional Supplementary Files [file 42003_2021_2697_MOESM2_ESM.pdf]

Description of Additional Supplementary Files

File name: Supplementary Data

Description: Supplementary Data 1. Gene family clustering in 14 species.

Supplementary Data 2. Enriched GO function for gene families expanded in T. yunnanensis. The p values were computed based on two-sided hypergeometric test and corrected with Benjamini-Hochberg. The q values were calculated using the Benjamini-Hochberg procedure to account for multiple testing.

Supplementary Data 3. The UniProt annotatin of 72 genes related to apoplast
Supplementary Data 4. Enriched GO function for gene families contracted in T. yunnanensis. The p values were computed based on two-sided hypergeometric test and corrected with Benjamini-Hochberg. The q values were calculated using the Benjamini-Hochberg procedure to account for multiple testing.

Supplementary Data 5. The genes unique to T. yunnanensis among T. yunnanensis, S. giganteum, G. montanum and G. biloba gene families.

Supplementary Data 6. Enriched KEGG pathway for unique gene families in T. yunnanensis. The p values were computed based on two-sided hypergeometric test and corrected with Benjamini-Hochberg. The q values were calculated using the Benjamini-Hochberg procedure to account for multiple testing.

Supplementary Data 7. Taxol biosynthesis metabolism genes.

Supplementary Data 8. The CYP725 genes in T. yunnanensis.

Supplementary Data 9. Summary of genes in the gene cluster on chromosome 12.

Supplementary Data 10. Summary of transcriptome data of T. yunnanensis.
