# Supplementary material for: Taxus yunnanensis genome offers insights into gymnosperm phylogeny and taxol production
Source: Commun Biol. 2021 Oct 20;4:1203. doi: 10.1038/s42003-021-02697-8 (PMC8528922; doi:10.1038/s42003-021-02697-8)
Supplement: Supplementary file 4 — Reporting Summary [file 42003_2021_2697_MOESM4_ESM.pdf]

nature portfolio

Corresponding author(s): Guibin Wang, Shilin Chen and Yves Van de Pee

Last updated by author(s): Jul 30, 2021

Reporting Summary

Nature Portfolio wishes to improve the reproducibility of the work that we publish. This form provides structure for consistency and transparency in reporting. For further information on Nature Portfolio policies, see our Editorial Policies and the Editorial Policy Checklist.

Statistics

For all statistical analyses, confirm that the following items are present in the figure legend, table legend, main text, or Methods section.

n/a	Confirmed	
☒	☐	The exact sample size (*n*) for each experimental group/condition, given as a discrete number and unit of measurement
☒	☐	A statement on whether measurements were taken from distinct samples or whether the same sample was measured repeatedly
☒	☐	The statistical test(s) used AND whether they are one- or two-sided<br>*Only common tests should be described solely by name; describe more complex techniques in the Methods section.*
☒	☐	A description of all covariates tested
☒	☐	A description of any assumptions or corrections, such as tests of normality and adjustment for multiple comparisons
☐	☒	A full description of the statistical parameters including central tendency (e.g. means) or other basic estimates (e.g. regression coefficient) AND variation (e.g. standard deviation) or associated estimates of uncertainty (e.g. confidence intervals)
☐	☒	For null hypothesis testing, the test statistic (e.g. *F*, *t*, *r*) with confidence intervals, effect sizes, degrees of freedom and *P* value noted<br>*Give P values as exact values whenever suitable.*
☒	☐	For Bayesian analysis, information on the choice of priors and Markov chain Monte Carlo settings
☒	☐	For hierarchical and complex designs, identification of the appropriate level for tests and full reporting of outcomes
☐	☒	Estimates of effect sizes (e.g. Cohen's *d*, Pearson's *r*), indicating how they were calculated

Our web collection on statistics for biologists contains articles on many of the points above.

Software and code

Policy information about availability of computer code

Data collection	No software had been used for data collection
Data analysis	SOAPnuke (v2.1.4), Oxford Nanopore GUPPY software (v0.3.0), STAR (2.5.1b;), HTSeq (v. 0.11.2) , GCEpackage (v.1.0.0), SMARTdenovo, Racon (v 1.4.11), Pilon (v1.22), Purge_haplotigs pipeline (v.1.0.4), ALLHIC(v0.9.12), Juicebox (v1.11.08), RepeatModeler (v.1.0.4), RepeatMasker (v.4.0.5), tBLASTn (V2.2.26), Minimap2 (v2.17), TransDecoder (v5.1.0), Augustus (v3.3.2), Genscan (v1.0), GlimmerHMM (v3.0.4), Exonerate (v2.4.0), MAKER (v 2.31.10), BUSCO software (v.4.1.2), Genewise (V24-1), BLASTP , InterProScan (v5.33), HMMER (v3.1), KOBAS (v3.0), OrthoMCL (v.2.0.9), MUSCLE (v.3.8.31), RAxML (v.8.2.10), Astral software (5.7.1), PAML (v.4.9), CAFÉ (v.2.1), BLASTp (V2.2.26), PAML package, MCScanX, MAFFT (v7.397) and RAxML (v8.2.10). Specific parameters used during run-time are provided in the methods. All softwares or scripts are available from official websites or GitHub as indicated in the methods.

For manuscripts utilizing custom algorithms or software that are central to the research but not yet described in published literature, software must be made available to editors and reviewers. We strongly encourage code deposition in a community repository (e.g. GitHub). See the Nature Portfolio guidelines for submitting code & software for further information.

Data

Policy information about availability of data

All manuscripts must include a data availability statement. This statement should provide the following information, where applicable:

- Accession codes, unique identifiers, or web links for publicly available datasets
- A description of any restrictions on data availability
- For clinical datasets or third party data, please ensure that the statement adheres to our policy

The data supporting the findings of this work are available within the paper and its Supplementary Information files. The data sets generated and analyzed during

this study are available from the corresponding author upon request. The genome sequence data and the transcriptome sequence data for T. yunnanensis have been deposited under NCBI BioProject number PRJNA661543.

Field-specific reporting

Please select the one below that is the best fit for your research. If you are not sure, read the appropriate sections before making your selection.

☒ Life sciences ☐ Behavioural & social sciences ☐ Ecological, evolutionary & environmental sciences

For a reference copy of the document with all sections, see nature.com/documents/nr-reporting-summary-flat.pdf

Life sciences study design

All studies must disclose on these points even when the disclosure is negative.

Sample size	No statistical methods were used to predetermine sample size. Our samples were all from wild type and did not use processed samples and groups.
Data exclusions	No data were excluded from the analyses
Replication	The genome sequence was taken and sequenced with more than 85 fold coverage. No replication is needed our genome reports.
Randomization	No random sampling is required for genome sequencing, because the genome differences are very small within the wild population, thus any wild plant is allowed for genome sequencing.
Blinding	We sequenced a single plant, and no control group is referred here. Blinding is not applicable in this study.

Reporting for specific materials, systems and methods

We require information from authors about some types of materials, experimental systems and methods used in many studies. Here, indicate whether each material, system or method listed is relevant to your study. If you are not sure if a list item applies to your research, read the appropriate section before selecting a response.

Materials & experimental systems

n/a	Involved in the study	
☒	☐	Antibodies
☒	☐	Eukaryotic cell lines
☒	☐	Palaeontology and archaeology
☒	☐	Animals and other organisms
☒	☐	Human research participants
☒	☐	Clinical data
☒	☐	Dual use research of concern

Methods

n/a	Involved in the study	
☒	☐	ChIP-seq
☒	☐	Flow cytometry
☒	☐	MRI-based neuroimaging
